# New geographic information system based sustainability metric for isolated photovoltaic systems

**DOI:** 10.1038/s41598-025-85222-9

**Published:** 2025-01-15

**Authors:** Rasha Elazab, Mohamed Daowd

**Affiliations:** Faculty of Engineering, Helwan University, Cairo, Egypt

**Keywords:** Isolated photovoltaic systems, Geographic information system, Dual-axis tracking PV, Solar irradiance variability, Sustainability metric, Climate change adaptation, Electrical and electronic engineering, Solar energy

## Abstract

The integration of photovoltaic (PV) technologies is vital for achieving sustainable energy solutions in isolated systems. However, A critical challenge that remains is maintaining the sustainability of these systems under the fluctuating conditions of solar irradiance, which is key for isolated energy systems. This study hypothesizes that the sustainability of PV systems can be accurately assessed through a new metric that incorporates performance consistency, variability, and resilience, using real-time energy production data alongside GIS-based solar radiation models. By analyzing fixed PV, concentrated PV (CPV), and dual axis tracking PV (DATPV) systems over a three-year period (2017–2019), The analysis indicates that DATPV systems achieved the highest energy output, with energy ratios exceeding 300% in 2019, though this was accompanied by substantial variability in performance. Fixed PV systems demonstrated the most stable performance, with a consistency term reaching 0.93 and a sustainability score of 0.87 in 2019. CPV systems performed moderately, with a sustainability score of 0.66 in 2017. These results highlight the trade-off between energy capture and operational stability, which is critical for sustainable energy management in isolated systems.

## Introduction

The concept of sustainability has evolved significantly, particularly in the context of energy systems^1^. According to^2^, sustainable development is defined as meeting the needs of the present without compromising the ability of future generations to meet theirs. However, over time, the definition of sustainable energy has often been oversimplified, focusing primarily on environmental protection using renewable sources such as solar and wind, while ignoring critical social and economic dimensions, especially for low-income communities^1^. Traditional sustainability frameworks frequently fail to address energy affordability and accessibility for vulnerable populations, leading to an imbalance between environmental goals and immediate energy survivability for these communities^3,4^.

Recent studies highlight the limitations of this global perspective, calling for a more localized approach to sustainability. As^3,5^ argue, energy availability plays a crucial role in poverty eradication and improving living standards, particularly in regions with limited access to modern energy services. This indicates a need for sustainability assessments that better consider the immediate energy needs of low-income communities, rather than solely focusing on future environmental benefits.

Recent advancements in PV systems have underscored the need for enhanced sustainability metrics, particularly in diverse environmental and operational contexts^6,7^. For instance, Chandel et al. (2024) analyzed the integration of PV-powered thermoelectric cooling systems for transitioning towards net-zero energy buildings under variable solar loading conditions, emphasizing the interplay between solar irradiance variability and system reliability^8^. Similarly, Tajjour et al. (2023) demonstrated how energy management systems could optimize the performance of PV microgrids, offering solutions for maximizing power generation under varying conditions^9^. These findings highlight the critical role of dynamic solar conditions and energy management in improving the sustainability of PV systems.

Studies have also explored geographical challenges, such as Tajjour et al. (2023), who investigated power generation enhancements in a grid-connected PV system in hilly terrains, where irradiance variability posed unique challenges^10^. Furthermore, the optimization of hybrid PV systems combining solar and wind resources in remote locations, as reviewed by Rawat and Chandel (2013), shows the importance of adaptability and efficient energy management^11^. These studies illustrate the need for sustainability metrics that account for irradiance variability for different PV systems.

Numerous studies have investigated the viability of solar photovoltaic (PV) systems, concentrating on sustainability dimensions such as technical, economic, and environmental aspects^12^. For instance^13^, explored the feasibility of using PV and Concentrating Solar Power (CSP) in copper mining in Chile, employing financial tools like benefit-to-cost ratio and internal rate of return (IRR). Similarly, studies on rooftop PV systems in industrial and commercial sectors in India showed strong economic feasibility, with payback periods as short as 3.72 years^14^. Additionally, research in the Middle East, such as Gaza and Qatar, has explored solar energy’s potential in addressing electricity shortages and integrating hybrid PV-SOFC systems in industrial applications^15,16^. In South Africa and Turkey, analyses demonstrated the economic and environmental benefits of large-scale PV systems, despite challenges like high initial costs^17,18^.

In Egypt^19^, highlighted the significant potential of solar PV, particularly in the educational sector, while studies in Saudi Arabia and China underscored the financial and energy-saving advantages of PV systems in industrial processes, particularly when integrated with dual-axis tracking systems^20,21^. Similarly, research in Pakistan revealed that grid-connected PV systems offered greater energy sustainability and economic benefits than off-grid systems, reducing energy demand by up to 35%^22^. In Morocco’s Souss-Massa Basin, PV systems were identified as a critical solution for water-energy-food (WEF) nexus challenges, especially in transitioning from fossil-fuel-based groundwater pumping^23^.

Although economic feasibility and environmental impacts of PV systems are well-documented, current sustainability metrics remain focused on these dimensions, often neglecting operational challenges associated with isolated PV systems. Most studies primarily assess grid-connected systems, which are inherently more reliable and efficient. However, the operational complexity of isolated systems—such as energy storage, reliability, and real-time energy management—remains underexplored, particularly for regions where grid connectivity is limited^24,25^. The deployment of isolated PV systems in such areas presents unique challenges that require new sustainability metrics, particularly those that assess the social impact, energy resilience, and operational adaptability of these systems. The variability in solar irradiance due to climate change further complicates system performance, yet this factor is often inadequately considered in traditional sustainability assessments^26^.

On the other hand, the Photovoltaic Geographical Information System (PVGIS) is a sophisticated, GIS-based tool that provides precise solar radiation estimates and PV energy production simulations. PVGIS calculates key irradiance values such as Direct Normal Irradiance (DNI), Global Horizontal Irradiance (GHI), and diffuse radiation, which are essential for a wide range of solar energy applications, including fixed PV, Concentrated Photovoltaic (CPV), and Dual-Axis Tracking PV (DATPV) systems. By integrating satellite-derived data with advanced meteorological models, PVGIS accounts for atmospheric variables like cloud cover, aerosol content, and elevation, ensuring highly accurate solar resource assessments for both fixed-axis and dual-axis tracking systems.

Several studies have utilized PVGIS models to improve the reliability of PV power generation^27^, short-term PV power forecasts^28^, and enhance PV system designs^29^. The versatility of PVGIS makes it a critical tool for researchers, policymakers, and system designers, enabling data-driven decision-making for renewable energy planning and system optimization. In this research, PVGIS is employed to generate daily solar irradiation data for each month under clear and cloudy sky conditions, offering a critical understanding of the solar energy potential and performance for PV, CPV, and DATPV systems^30^.

This study introduces a novel sustainability metric that quantifies the impact of climate change by comparing Geographic Information System (GIS)-based solar radiation models with real-world solar radiation data across three distinct years. This comparison helps capture how well the system can adapt to climate variability over time.

**Fig. 1:**
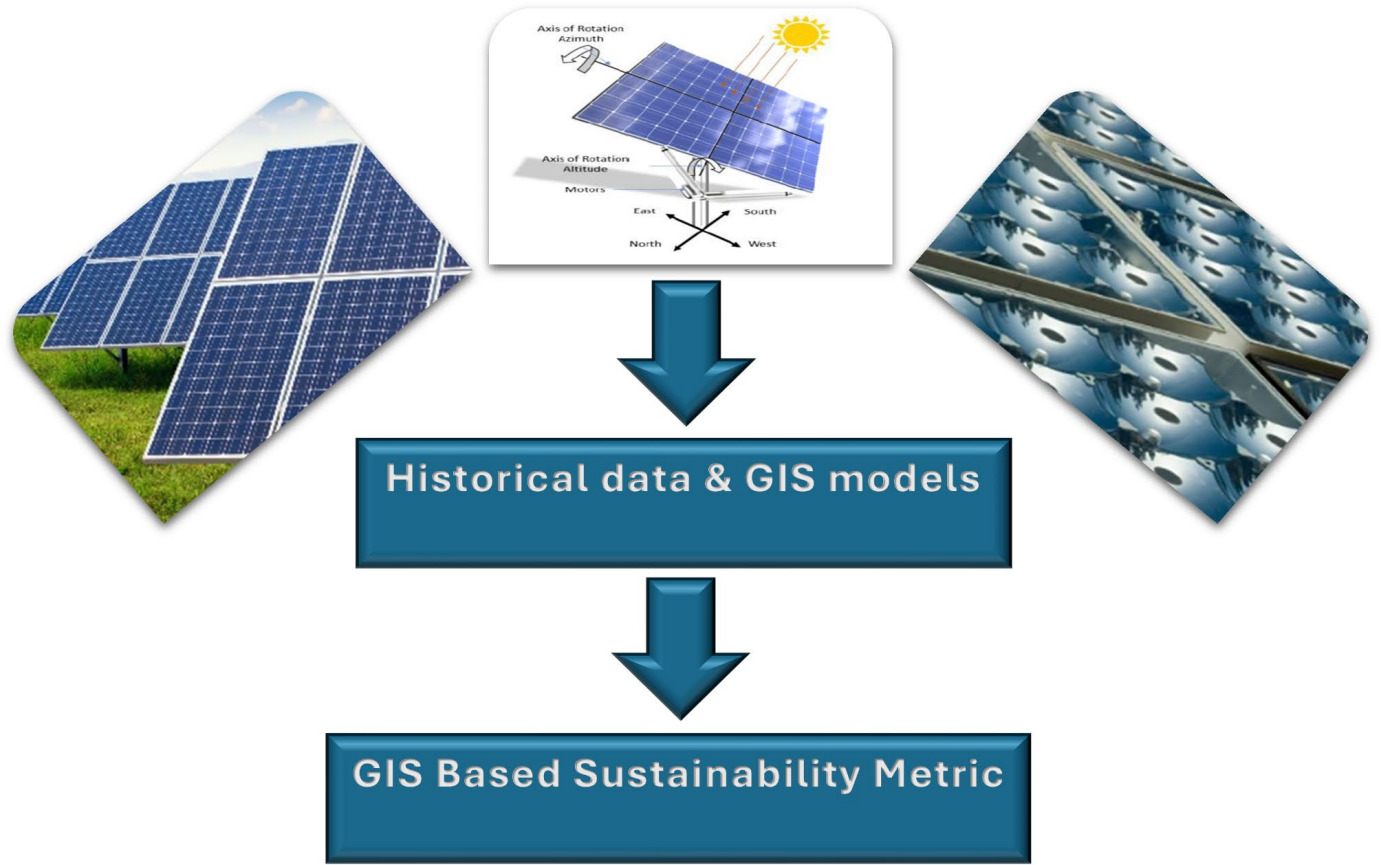
The conceptual framework of the proposed metric.

Solar irradiance variability, driven by changing weather patterns and long-term climate change, can influence the performance and reliability of PV systems. By analyzing discrepancies between modeled and actual solar radiation, this metric aims to capture the system’s ability to adapt to climate variability and ensure consistent energy output, as shown in Fig. 1.

The main contributions can be summarized as follows:

- This study introduces a new sustainability metric that combines performance consistency, and variability into a single comprehensive score.
- Unlike previous approaches that analyze these factors separately, the proposed metric offers an integrated assessment of PV system sustainability.
- The metric focuses on the comparison between real energy production and GIS-modeled energy production, providing a more accurate understanding of system performance.
- This approach introduces a new dimension to sustainability evaluations by assessing how closely real-world performance aligns with modeled expectations over time.
- The proposed metric enhances the ability to make informed decisions regarding the planning and management of isolated renewable energy systems under varying solar conditions.

This paper is structured as follows: Sect. 2 introduces the methodology used for developing the new sustainability metric, including the integration of GIS models with historical solar irradiance data. Section 3 presents a detailed case study of PV, CPV, and DATPV systems in Cairo, Egypt, across three years, highlighting the performance variability under different solar conditions. Section 4 discusses the results of the sustainability analysis, comparing the consistency, variability, and overall sustainability metrics of the three PV systems. Finally, Sect. 5 concludes the paper by summarizing the key contributions and outlining potential future work aimed at improving the sustainability of isolated PV systems under varying climatic conditions.

## GIS based sustainability metric

A climate change sustainability criterion for isolated systems should prioritize two key factors: consistency, and resilience. Consistency refers to the ability of the system to maintain energy production close to the expected daily energy output. Resilience involves the system’s capacity to handle variability beyond the modeled energy during the year, particularly in response to unpredictable climatic shifts. The proposed sustainability metric can be formulated as a weighted combination of the system’s long-term performance consistency, the deviation from expected performance based on GIS-based models, and a resilience factor that accounts for energy output variability. This metric offers a comprehensive assessment of a system’s sustainability by integrating both modeled expectations and real-world performance under varying conditions.

The Sustainability Metric (SM) is a weighted score that combines the long-term consistency of system performance, the deviation from GIS-modeled energy outputs, and a variability factor that captures daily fluctuations in energy production. The formula is expressed as

1 $$SM = \left( {1 - \frac{1}{N}\sum\limits_{{i = 1}}^{N} {\frac{{\left| {R_{i} - 100\% } \right|}}{{100\% }}} } \right) \times \left( {1 - V} \right)$$

*Where: N: Total number of days in the dataset (365 for a full year)*,* R*_*i*_: *Ratio of real energy production to GIS-modeled energy production on day i*,* expressed as a percentage*,* and V: Variability factor of daily ratios R*_*i*_*over the year.*

The performance consistency term is designed to evaluate how closely the system’s actual energy production aligns with the ideal performance, which is represented as 100% of the expected output based on GIS models. This term is calculated by averaging the absolute deviations between the real energy production and the modeled value over a period of *N* days. The smaller the deviation from the ideal 100%, the higher the performance consistency. A lower deviation reflects a system that consistently produces energy in line with the modeled expectations, which is crucial for ensuring consistent performance in isolated PV systems.

The variability factor (V) quantifies the degree of fluctuation in daily energy production by calculating the ratio of the standard deviation (σ) to the mean (µ) of the daily production ratios. This factor captures the day-to-day variability in the system’s performance, with higher variability indicating greater instability in energy output. A system with high variability will exhibit significant deviations from its average energy production, which is undesirable in isolated PV systems that require stable, predictable energy generation.

As such, higher variability results in a lower sustainability score, emphasizing the importance of minimizing fluctuations for long-term system resilience. The closer the *SM* value is to 1, the more sustainable the system is, meaning it consistently produces energy close to the expected amount, with minimal variability. Systems with high fluctuations (high ) and large deviations from expected performance will have a lower SM.

## Case study

**Fig. 2:**
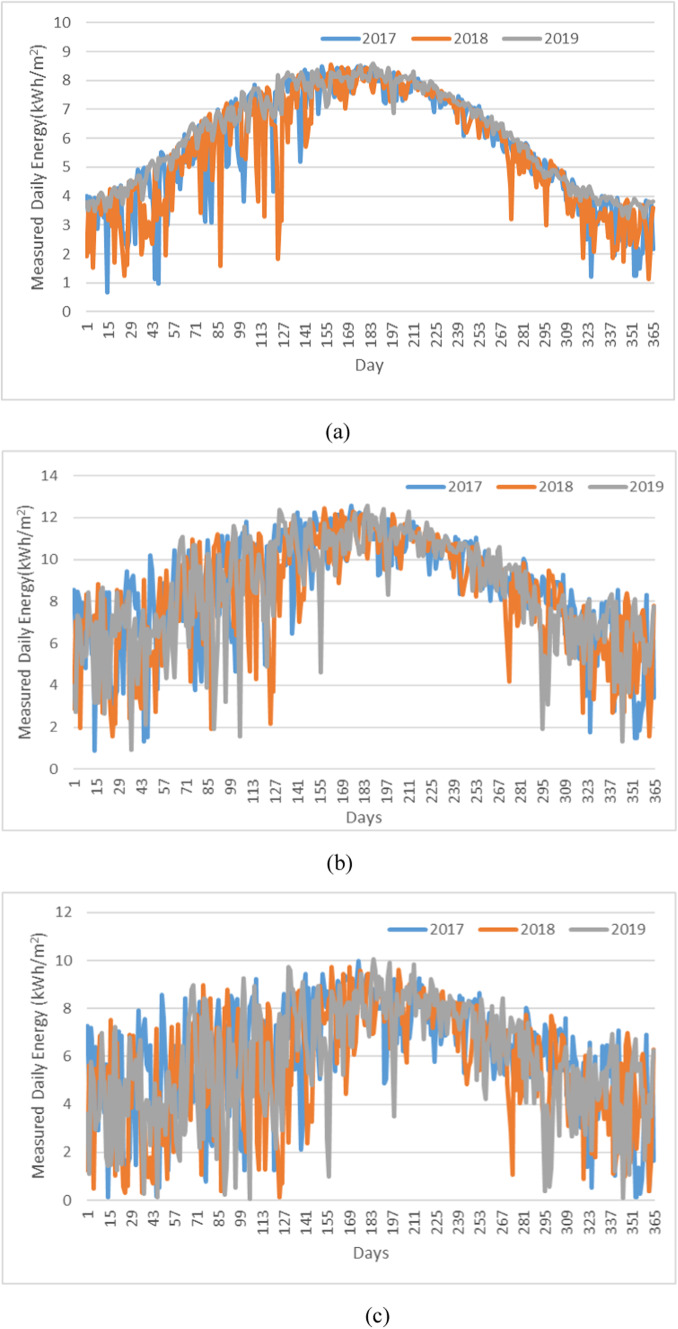
Measured daily energy : **a** PV, **b** DATPV, **c** CPV.

Cairo, Egypt (30.0444°N, 31.2357°E), is examined. Cairo is characterized by high solar irradiance levels, making it an ideal location for solar energy harvesting. This location’s semi-arid climate provides a mixture of clear skies and intermittent cloud cover, allowing for a comprehensive analysis of solar radiation variability throughout the year. Historical data for different solar irradiances spanning 12 months was compiled for the selected study locations for three different years, i.e. 2017–2019, as referenced^31^. Figure 2 shows the studied historical daily energy behaviours for different PV systems.

**Fig. 3:**
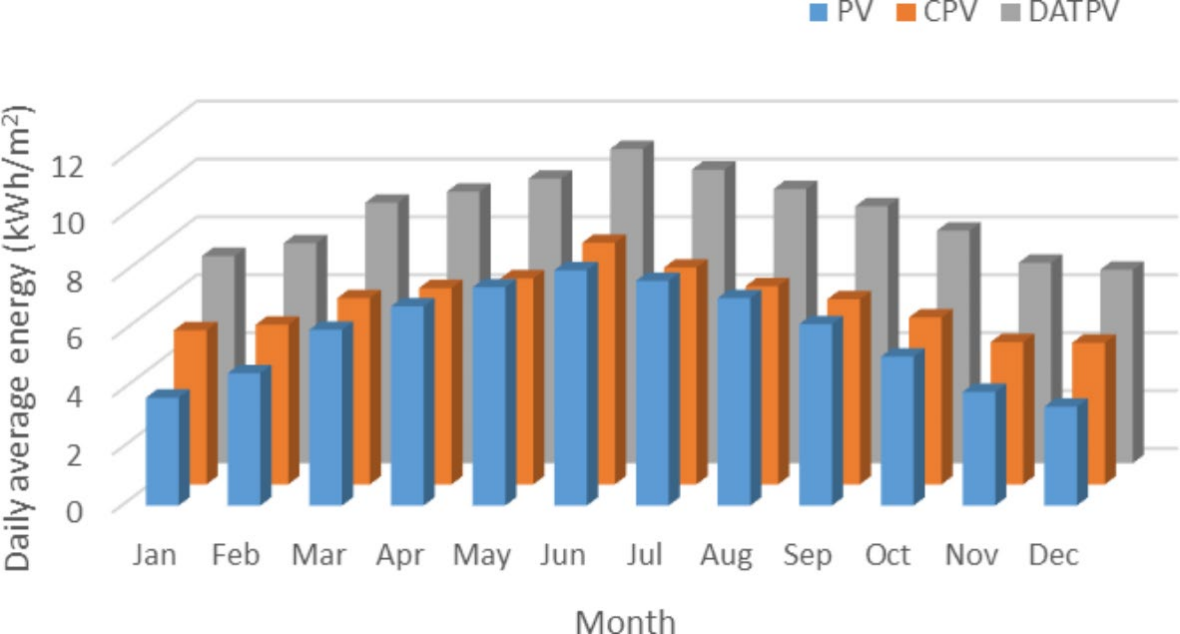
GIS based average daily energy for different PV systems.

Historical data show large variations from year to year, driven by unpredictable climate events or anomalies, which complicates accurate forecasting of future performance. Moreover, the increasing effects of climate change make it harder to predict future irradiance patterns based solely on past trends. therefore, relying only on historical data can be enormously confusing. Figure 3 illustrates the average energy (kWh/m²) output for the PV, CPV, and DATPV systems on a monthly basis across the year.

DATPV systems show the highest energy output throughout the year, with especially strong performance in the summer months (June to September). This aligns with Fig. 2 showing a higher daily real energy relative to other systems. The system’s tracking mechanism clearly maximizes solar energy capture, particularly when solar irradiance is at its peak during the summer. CPV systems are lower than DATPV but consistently outperform the fixed PV system across the year. This is expected, given the concentrated photovoltaic system’s ability to focus sunlight onto a smaller area, increasing its energy production. While fixed PV systems show the lowest monthly energy output, reflecting the absence of any tracking or concentrating technology. The system performs more consistently across the year but lacks the ability to significantly increase energy production during peak solar months.

On the other hand, while radiation models like GIS-based solar radiation models and average daily energy models provide valuable estimates, they often fail to capture the full variability of solar irradiance due to factors such as weather patterns, seasonal changes, and localized environmental conditions. This limitation can lead to significant discrepancies between modeled performance and actual system behavior. As a result, relying solely on these models can be equally misleading, especially when comparing the sustainability of different PV systems, as they may not fully reflect the operational challenges and real-world performance of these systems over time.

These challenges highlight the need for a new criterion to assess the sustainability of PV systems. A more consistent metric would integrate both historical data and radiation models, analyzing irradiance patterns to ensure a realistic and sustainable evaluation. Given that solar irradiance is the primary driver of PV power generation, such a criterion would better account for radiation variability and offer a clearer picture of the long-term sustainability of PV systems, especially for isolated applications where consistent energy output is critical.

## Results and discussion

**Fig. 4:**
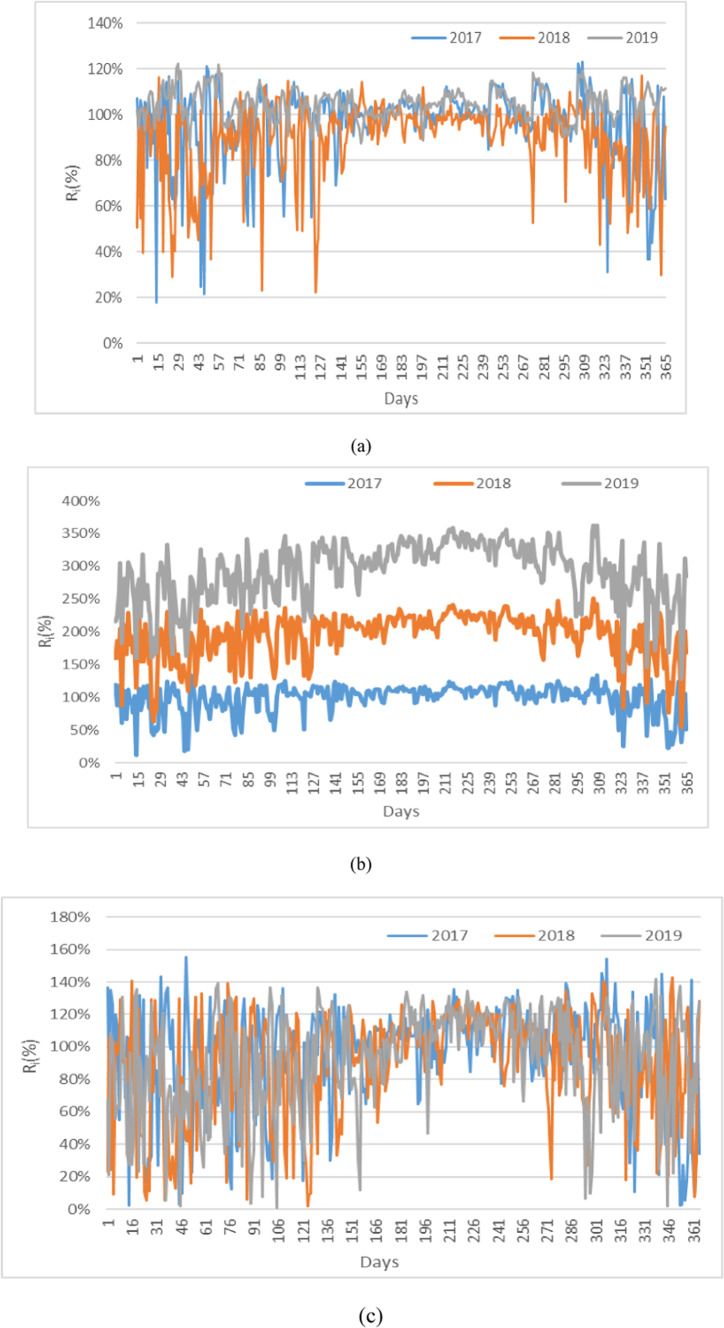
Daily *R*_*i*_ ratios : **a** PV, **b** DATPV, **c** CPV.

A comprehensive analysis is presented of the ratios between actual daily solar energy production and the modeled values *R*_*i*_, which serve as key indicators of the systems’ performance under varying solar irradiance conditions. These ratios, illustrated in Fig. 4, provide insights into the efficiency and variability of different PV technologies, such as fixed PV, CPV, and DATPV, over multiple years. By examining these ratios, the section highlights how each system responds to real-world fluctuations in solar irradiance and evaluates their sustainability, reliability, and suitability for isolated energy systems.

Figure 4 demonstrates that the DATPV system in 2019 exhibited the highest variability in daily energy ratios, surpassing 300%, compared to lower variability in 2017 and 2018. This indicates that DATPV’s ability to track the sun throughout the day significantly enhanced energy capture, particularly in 2019. In contrast, the energy ratios for both fixed PV and CPV systems were lower, with CPV showing fluctuations between 0% and 180%, while fixed PV remained more stable, generally close to 100%.

Throughout the three years, DATPV consistently produced more energy compared to CPV and fixed PV systems. However, its higher variability, particularly in 2019, underscores a trade-off between maximizing energy capture and maintaining consistent output, especially critical for isolated systems. In contrast, the more stable performance of PV and CPV systems offers better predictability for energy storage and battery management, crucial for ensuring reliability in isolated PV systems.

The higher energy output DATPV systems, as shown in Fig. 4, results from their ability to dynamically adjust to the sun’s position across both azimuth and elevation angles. This feature allows DATPV systems to maximize energy capture, especially in regions like Egypt, which are characterized by high solar irradiance and minimal cloud cover. However, the sustainability of DATPV systems is not uniform across all geographical areas. In regions with frequent cloud cover, lower solar angles, or significant seasonal variation, the variability in daily energy production may outweigh the benefits of increased energy capture. For example, the Egyptian case demonstrates a clear advantage for DATPV systems in energy output but also highlights increased variability, with sustainability metrics for DATPV scoring lower than those of fixed PV systems due to higher fluctuations. These findings underscore the importance of region-specific analysis when determining the most sustainable PV technology, as local climatic and operational factors significantly influence system performance and reliability.

The results provide a detailed comparison of the performance of PV, DATPV, and CPV systems across three years (2017, 2018, and 2019), using three key metrics: consistency term, variability term, and the overall sustainability metric, as shown in Table 1.

**Table 1:** Sustainability analysis metrics.

Metric	PV			DATPV			CPV		
	2017	2018	2019	2017	2018	2019	2017	2018	2019
Consistency term	0.89	0.87	0.93	0.77	0.74	0.75	0.84	0.83	0.84
Variability term	0.83	0.81	0.94	0.67	0.63	0.66	0.78	0.76	0.78
Sustainability metric	0.74	0.71	0.87	0.52	0.46	0.49	0.66	0.63	0.65

The consistency term highlights how closely each system’s real energy production aligns with the modeled energy output. Fixed PV demonstrates the highest consistency across all years, particularly in 2019 (0.93), indicating a stable and predictable performance. DATPV shows lower consistency, reflecting greater fluctuations due to its dependence on dynamic solar tracking, with 2019 scoring slightly higher (0.75) than previous years. CPV remains moderately consistent across all years, with values around 0.84.

The variability term measures day-to-day fluctuations in energy output. Here, fixed PV once again outperforms DATPV and CPV, particularly in 2019 (0.94), underscoring its stability. DATPV exhibits the highest variability, particularly in 2018 and 2019, which can be attributed to its more sensitive tracking mechanisms. CPV’s variability remains between that of fixed PV and DATPV, indicating it has better control over fluctuations than DATPV but is less stable than fixed PV.

The overall sustainability metric combines these factors, and the results show that fixed PV systems consistently rank the highest in sustainability, with 2019 reaching 0.87, reflecting its stable performance and low variability. DATPV, while capable of higher energy capture, struggles with variability and consistency, resulting in the lowest sustainability scores, particularly in 2018 (0.46). CPV systems, with moderate consistency and variability, score mid-range in sustainability, peaking at 0.66 in 2017.

In conclusion, while DATPV offers potential for higher energy output, its variability reduces its overall sustainability, especially in isolated systems. Fixed PV demonstrates the best balance between performance stability and reliability, making it the most sustainable option based on these metrics. CPV provides a middle ground, offering a mix of energy capture potential and moderate stability.

## Conclusion

This study presents a comprehensive analysis of the sustainability of different PV technologies for isolated energy systems, using a newly developed metric that integrates performance consistency and variability. The findings show that while DATPV systems capture the highest energy output, their sustainability is compromised by significant fluctuations, with variability terms as low as 0.63 in 2018. In contrast, fixed PV systems offer the most reliable performance, achieving a consistency term of 0.93 and a sustainability score of 0.87 in 2019, making them the most suitable for isolated systems requiring steady energy output. CPV systems demonstrate moderate performance, with a balance between energy production and stability, peaking with a sustainability score of 0.66 in 2017. These results underline the importance of evaluating not just energy output but also the consistency and adaptability of PV systems to variable solar conditions. The proposed sustainability metric provides a more comprehensive understanding of system performance, allowing for more informed decision-making in the planning and management of isolated renewable energy systems. Future work will explore the integration of battery sustainability and advanced energy management systems to further enhance the sustainability of isolated PV systems, ensuring optimized energy storage and distribution under variable solar conditions.

Future research should focus on refining the proposed sustainability metric to incorporate the impact of energy storage technologies. Investigating the integration of hybrid renewable energy systems, such as PV-wind or PV-biomass combinations, could provide a more holistic perspective on sustainability, particularly for isolated systems. Moreover, applying the metric to a broader range of geographical locations and climatic conditions would validate its adaptability and robustness.

## Data Availability

The datasets used and generated during the current study are available from the corresponding author upon reasonable request.
