# Publisher Correction: New geographic information system based sustainability metric for isolated photovoltaic systems

**DOI:** 10.1038/s41598-025-92483-x

**Published:** 2025-03-18

**Authors:** Rasha Elazab, Mohamed Daowd

**Affiliations:** Faculty of Engineering, Helwan University, Cairo, Egypt

Correction to: *Scientific Reports* 10.1038/s41598-025-85222-9, published online 15 January 2025

The original version of this Article contained an error in Figure 1 where the text was replaced by symbols. The original Figure 1 and accompanying legend appear below.

**Fig. 1:**
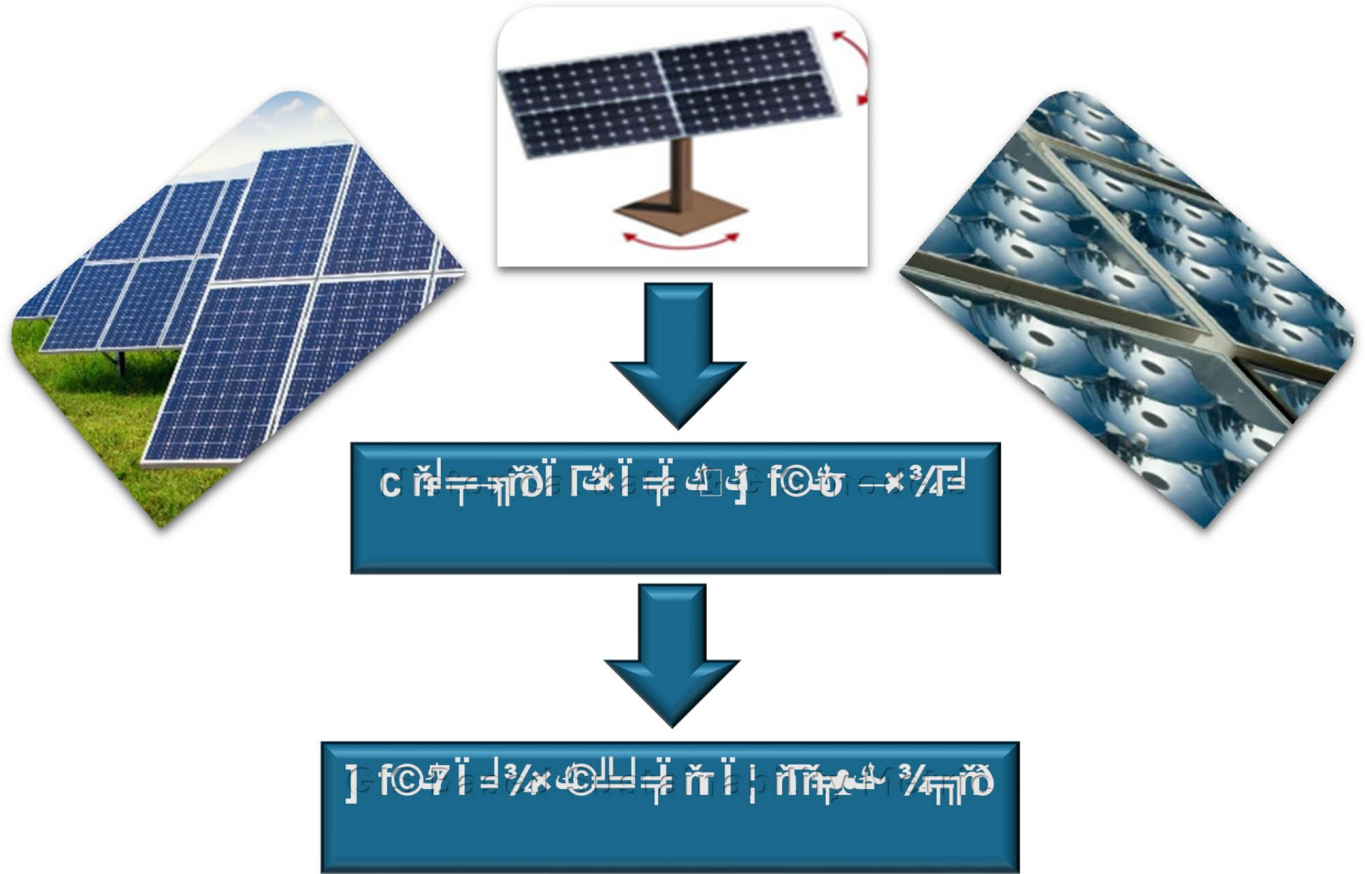
The conceptual framework of the proposed metric.

The original Article has been corrected.

